# Influence of needle working length and root canal curvature on irrigation: a computational fluid dynamics analysis based on a real tooth

**DOI:** 10.1186/s12903-022-02205-2

**Published:** 2022-05-14

**Authors:** Na Zhou, Zhengqiu Huang, Mingzhou Yu, Shuli Deng, Baiping Fu, Hanhui Jin

**Affiliations:** 1 Stomatology Hospital, School of Stomatology, Zhejiang University School of Medicine, Clinical Research Center for Oral Diseases of Zhejiang Province, Key Laboratory of Oral Biomedical Research of Zhejiang Province, Cancer Center of Zhejiang University, 310006 Hangzhou, People’s Republic of China; 2 College of Mechanical and Electrical Engineering, China Jiliang University, 310006 Hangzhou, People’s Republic of China; 3 School of Aeronautics and Astronautics, Zhejiang University, 310006 Hangzhou, People’s Republic of China

**Keywords:** Root canal curvature, Needle working length, Flat-needle, Computational fluid dynamics analysis, Irrigation flow

## Abstract

**Backgrounds:**

To compare the irrigation efficiency with different needle working length and different root canal curvature based on a real unshaped root canal using computational fluid dynamics (CFD) method.

**Methods:**

Images of the root canal of the maxillary first molar after being prepared to .04/15 were scanned using micro-CT, and then imported into the software for three-dimensional reconstruction. A palatal root canal with a curvature of 23.4° was selected as the experiment canal. The needle working length of the 30-G flat needle was 4.75 mm, 5 mm, 5.25 mm and 5.5 mm short of apical foramen respectively, the flow pattern, irrigation velocity, shear stress were compared. The modified curved canals with a curvature of 0°, 5°, 10°, 20° and 30°were reconstructed via software. The flat needle was replaced at the optical inserted depth, and key parameters of irrigation efficiency were analyzed.

**Results:**

Decreased needle working length had a positive impact on irrigation efficiency. With the optimal needle working length, the replacement of the apical irrigation fluid, the effective velocity, and wall shear stress were significantly improved in more severely curved root canals. With the same needle working depth and analogous canal curvature, irrigation efficiency is higher in real canal than that of modified canal.

**Conclusions:**

Short needle working depth, large curvature and the anomalous inner wall of canals help to improve irrigation efficiency.

## Background

The main approach to root canal therapy is to remove the infected dental pulp and the smear layer through biomechanical treatment and root intracanal irrigation [1, 2]. Root canal irrigation has great advantages in the removal of bacteria, debris, necrotic tissues and smear layer in areas unreachable by mechanical instruments, including isthmus, fins and lateral canals [3, 4].

The flow field generated in the root canal basically determines the final performance of irrigation. Computational fluid dynamics (CFD) has been regarded as a powerful method in the exploration of root canal irrigation. Studies have explored the effect of factors such as the inflow velocity of the irrigation [5], the size of the root canal preparation [6], the needle type [7], the taper of the root canal [8], the needle-insertion depth [9], and the irrigation temperature on irrigation [10]. The critical shear stress is the least shear stress that peels off the smear layer on the root canal wall and has been proposed in the evaluation of the cleanse ability of the flow field [10]. Nevertheless, the root canal models applied in these studies were simple artificial root canal models composed of a straight conical pipe with a taper. They are far different from the actual human root canal anatomy with bending, convex, concave and anomalous walls [11–13]. Curvature is one of the main shape parameters. Particularly, the curvature in the root canal of the various real teeth can differ from each other [14, 15]. Also, the difference in the curvature of the root canal potentially exerts significant effects on the preparation, irrigation, and obturation of root canals [16–18]. With a simple artificial tapered root canal model, the results can inevitably lead to inaccurate conclusions, among them, overestimated or underestimated irrigation effect and debris removal ability [19]. However, only a few computational studies of irrigation have been performed using the real human root canal, and none of the studies reported on the effect of the canal curvature [19–21]. In previous literature reports, researchers used clinical trials or experimental devices to evaluate the irrigation performance of root canals with different curvatures [22, 23].

In this article, we employed micro-computed tomographic technology to obtain the model of real human root canal anatomy with complex characteristics [24, 25]. Using the digital image, the flow field inside the real root canals was numerically investigated during irrigation. For CFD studies on the irrigation of a real root canal, a flat needle was inserted at different depths was used for numerical simulation. Lastly, we explored the influence of canal curvature on irrigation.

## Methods

### Root canal irrigation model

This study was approved by the Ethics Committee of the Affiliated Hospital of Stomatology, Zhejiang University (Certificate 2018-030). We collected extracted maxillary first molars with complete roots and mature apexes. The dental remnants and soft tissue were removed from the root surface, and then the teeth were stored in 0.1% thyme solution at 4 ℃ for subsequent use. Micro-CT scanning (Scanco-Medical micro-CT 100 system; Scanco Medical, Bassersdorf, Switzerland) was employed to select a tooth with a separate palate root and the curvature of 23.4^°^.

**Fig. 1:**
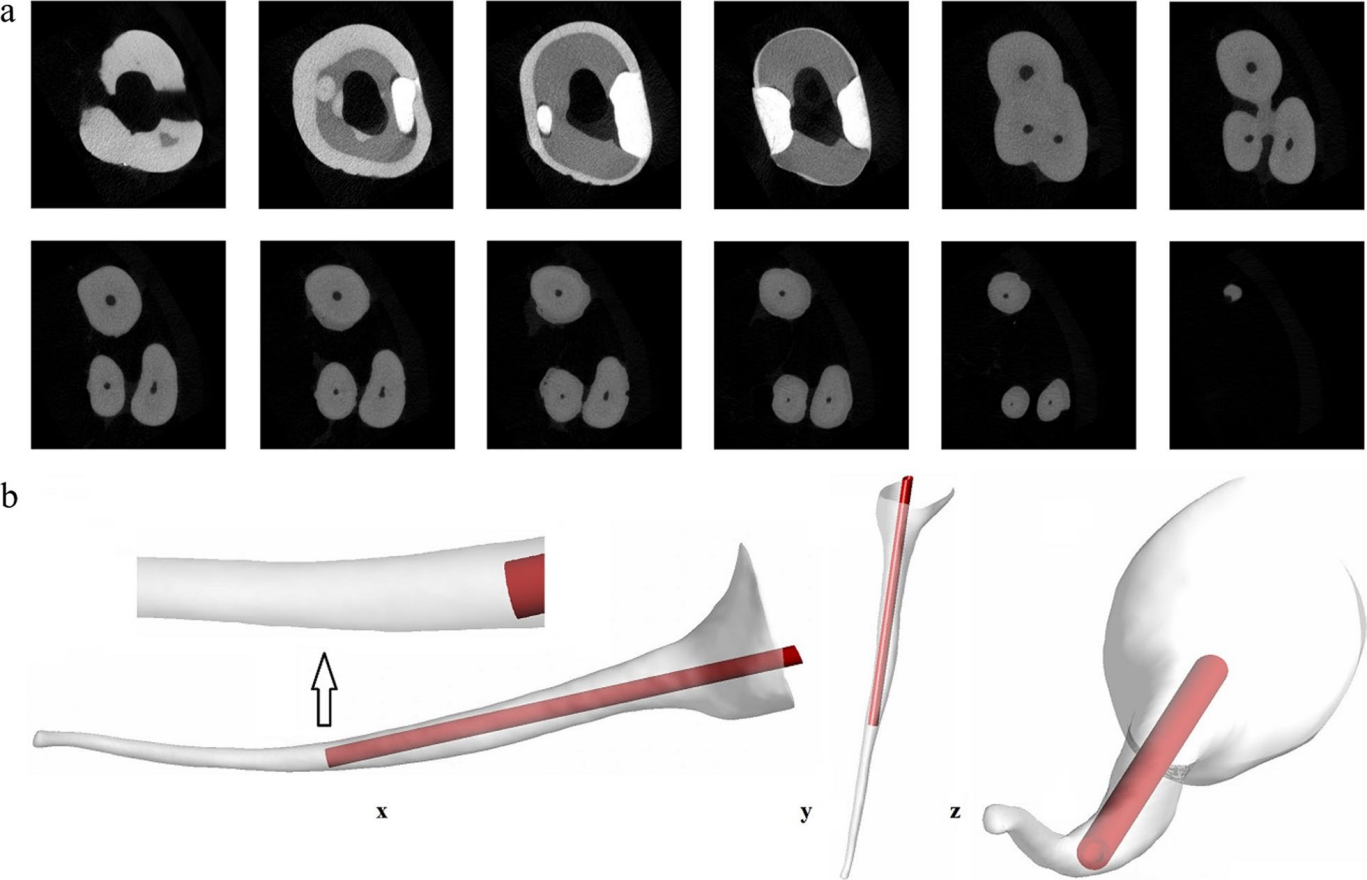
**a** Micro-CT scanning from a maxillary first molar; **b** geometrical root canal model with a 30-G flat needle with x, y, and z positions

Instrumentation and shaping of the palate canal were conducted using a size 15/04 Profile Vortex Blue file (Dentsply Tulsa Dental Specialties, Tulsa, OK) up to the work length. Following the micro-CT scanning setting: 30-μm istropic resolution, energy of 90 kV, and current of 200 μA, and 500 ms integration time, 685 transverse slice images (Tiff format) at different cross-sections of the root canal were obtained using the micro-CT system (Fig. 1a). Using the cross-section images, a 3-dimensional real root canal model was then reconstructed [26]. SolidWorks 2019, (Dassault Systemes, Waltham, MA), a 3D CAD design software, was used for quantitative measurements of the canal diameter, the reconstruction real root canal model had a verified apical size of 0.15 mm. The most frequently used 30-G flat needle, was adopted in this experiment. The sizes of the needle were standardized based on the external diameter (D_ext_), the internal diameter (D_int_) and the length (L) as follows: D_ext_ = 320 μm, D_int_ = 196 μm and L = 31 mm, respectively. The contour of streamlines was investigated in x, y, z positions (Fig. 1b).

**Fig. 2:**
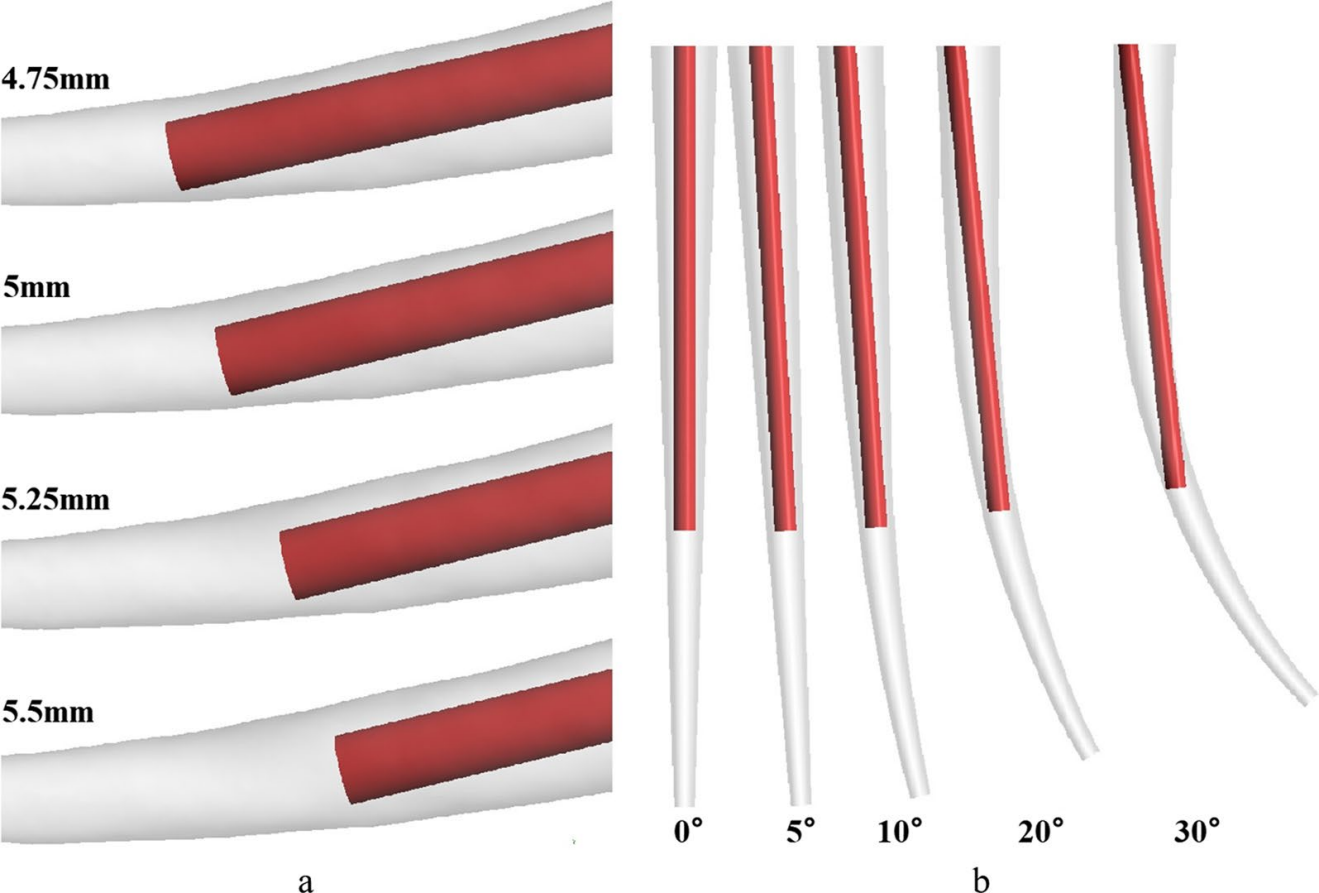
**a** The root canal irrigation model with different needle working depth; **b** the root canal irrigation model with different curvature

To ensure the irrigant can flow into the root canal smoothly, 0.01 mm of space distance between the flat needle tip and inner wall of root canal was maintained. Therefore, the least needle working length was 4.75 mm short of work. To establish the effect of the needle working length on the cleanse efficiency, inserted depths 4.75 mm, 5 mm, 5.25 mm and 5.5 mm short of root canal work length were selected, respectively (Fig. 2a). Further, 910,000 computational unstructured tetrahedral meshes were arranged to conduct CFD simulation.

To explore the influence of the bending curvature, more root canal models were erected on the basis of the obtained real canal root model. Based on the inserted depth yielding optimal irrigation, the bending curvature was replaced, in inner wall in the root canal model was smoothed and optimized, whereas other parameters remained constant [25]. According to the common clinical root canal curvature distribution range [14], bending curvatures of 0°, 5°, 10°, 20° and 30° were selected at 4.75 mm of needle working length (Fig. 2b).

### Boundary conditions and numerical simulation

The numerical simulation was completed using the commercial software Ansys Fluent 18.1 (ANSYS Inc, Canonsburg, PA, USA). The irrigation fluid was set as an incompressible, Newtonian fluid with a density of 1.04 g/cm^3^ and the viscosity of 1.3 × 10^–3^ Pa*s [27]. The surfaces of the root canal and the needle were set as a rigid, impermeable, and non-slip boundary. The gravity acceleration of the ambient environment was 9.8 m/s^2^. The k-ω SST turbulence model for simulation as described in previous works was adopted [19]. The averaged inflow velocity of 8.6 m/s in the axial direction was set as the inlet boundary condition, which was equivalent to the flow rate of 0.26 mL/s in actual irrigation [5].

The replacement of the local fluid flow, the shear stress, and the average pressure at the apical root canal served as the characteristic parameters for irrigation efficiency. Clean depth, is the irrigant touch the canal wall in long axis direction, and clean span is the irrigant touch the canal wall in the cross range. Clean depth and clean span are calculated as mm, representing the maximum vertical and horizontal magnitude of the flushing fluid. The effects of the needle working length and the curvature on the irrigation process at different inflow velocities were explored. A magnitude of > 0.1 m/s is regarded as the effective velocity that can effectively replace and transport the polluted irrigant with the fresh irrigant [5, 6]. The critical shear stress, that is, the lowest shear stress required to peel off the smear layer on the root canal wall, was considered to be 100 Pa at normal temperature [10].

## Results

### Effect of needle working length on irrigation flow in the real root canal model

**Fig. 3:**
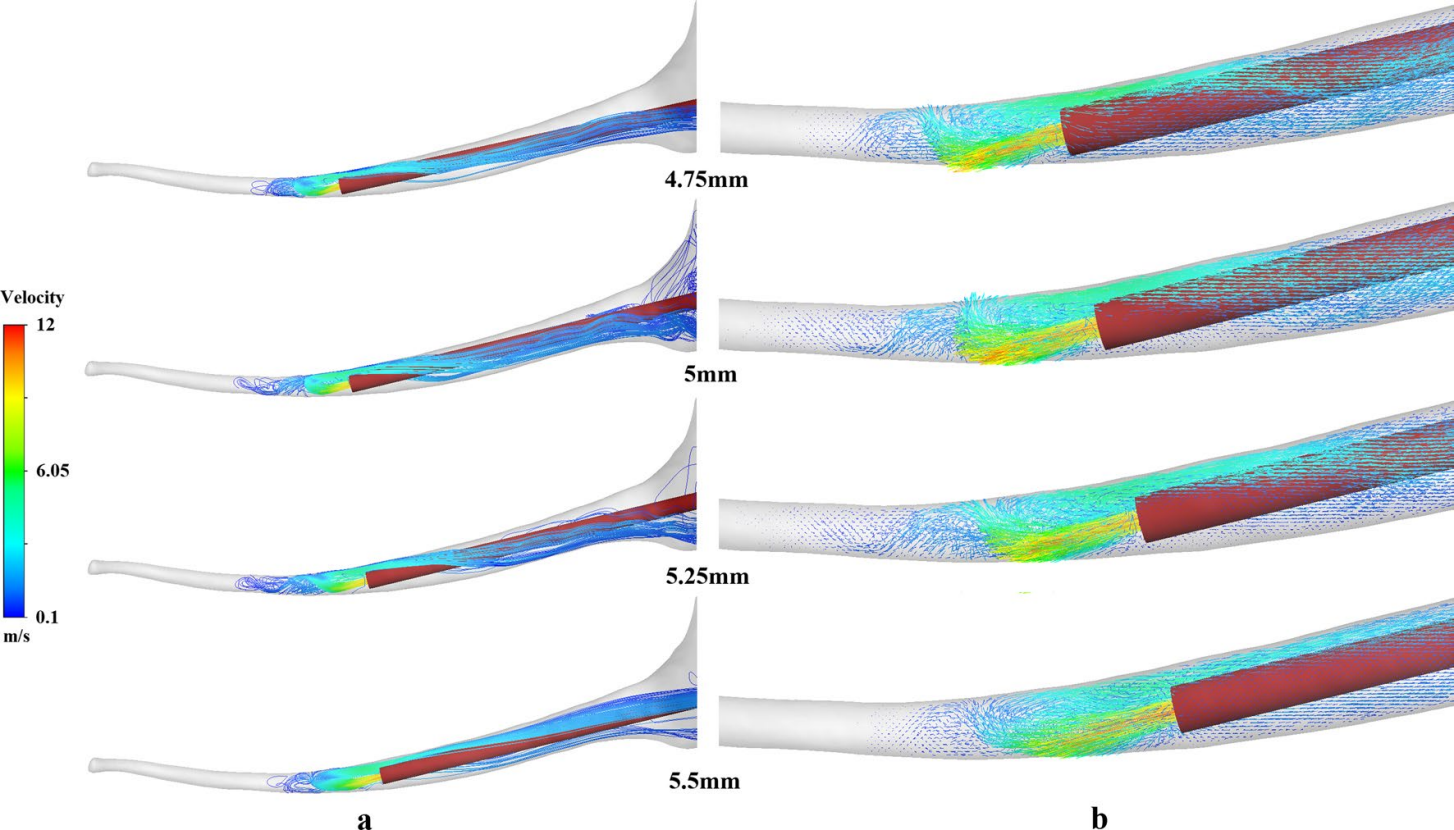
Effect of needle working length on the irrigation flow field. **a** Streamlines; **b** velocity vectors

Figure 3 shows the velocity vectors of the flow field near the flat needle tip during irrigation. Due to the curving of the real root canal, the irrigation fluid flushed the wall facing to the tip first, then directed the flow to the opposite side of the wall. Eventually, the circulation zone was organized. The flush flow velocity decreased continuously then stagnated to generate the dead water zone at the apical root canal. The stagnant dead water zone proved unfavorable to the replacement of the polluted irrigant. At different needle working length the flat needle, the streamlines in the flow field revealed a clean depth (Fig. 3a). The needle working length, 5 mm and 5.25 mm showed noticeable advantages in the clean depth over other needle working length. Meanwhile, the distribution of the velocity vectors demonstrated that the change of the needle working length could alter the configuration of the flow field near the needle tip (Fig. 3b). With the alternation of the flow field, the needle working length may influence the clean depth, the clean ability of the root canal and the apical pressure.

Table 1 outlines the maximum depth attained under the effective velocity at different needle working length. Of note, effective clean depth under velocity increased to about 9.75 mm with an increase in the needle working length from 4.75 mm to 5 mm. Also, Table 1 shows the effect of needle working length on the clean depth and the clean span of the effective shear stress on the root canal wall. The clean depth increased with the shortening needle working length. Both the clean depth and the clean span attained the maximum value at the needle working length of 4.75 mm. Besides, a large span could be obtained from the working length of 5.25 mm. Thus, an appropriate inserted depth is essential for an optimized clean efficiency.

**Table 1:** Effect of needle working depth on irrigation efficiency in a real 23.4°-curved-root canal with a 30-G flat needle

Needle working length (mm)	Depth of effective velocity^*^ (mm)	Effective shear stress^#^	
		Clean span (mm)	Clean depth (mm)
4.75	9.50	4.96	9.04
5	9.75	4.69	9.01
5.25	9.69	4.84	8.78
5.5	9.08	4.65	8.59

*Effective velocity: > 0.1 m/s; ^#^effective shear stress: ≥ 100 Pa

**Fig. 4:**
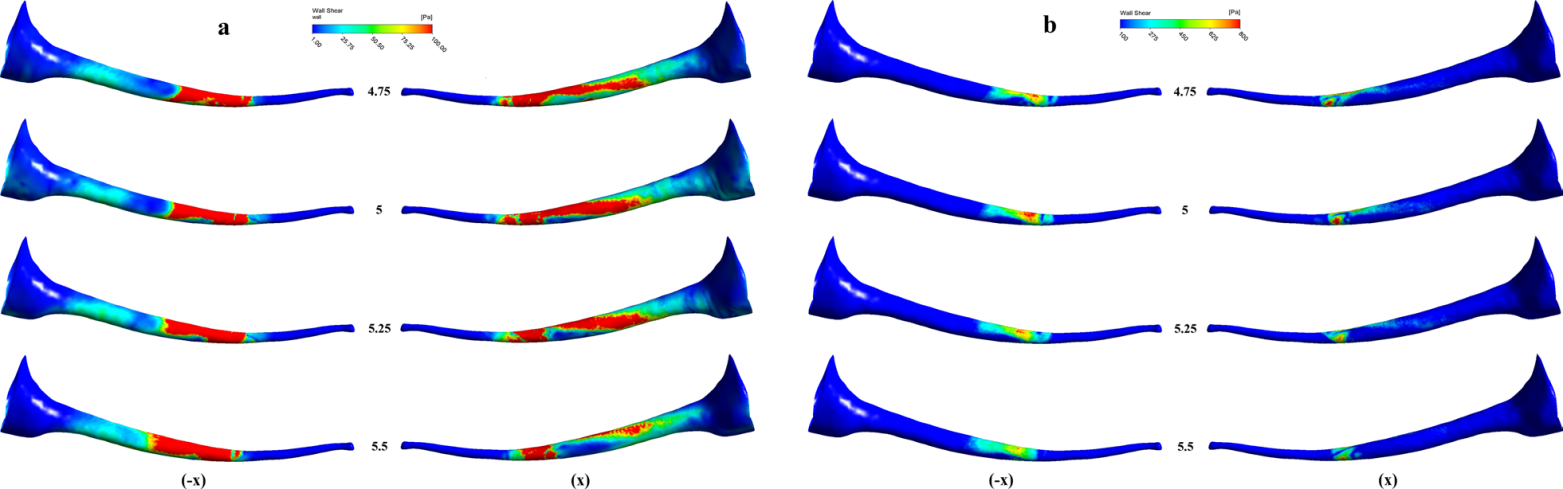
Effect of needle working length on shears tress, displayed in the -x and x directions respectively **a** 0–100 Pa; **b** 100–800 Pa

The contour of shear stress on the wall of the root canal is shown in Fig. 4. To assess the distribution of the shear stress, higher than the critical shear stress, the contours of shear stress 0–100 Pa and > 100 Pa were displayed in the -x and x directions (namely the positive and negative directions perpendicular to the paper plane), respectively, The needle working length could influence the distribution and coverage of the effective shear stress.

### Effect of different root canal curvatures on irrigation flow in the optimized canal model

**Fig. 5:**
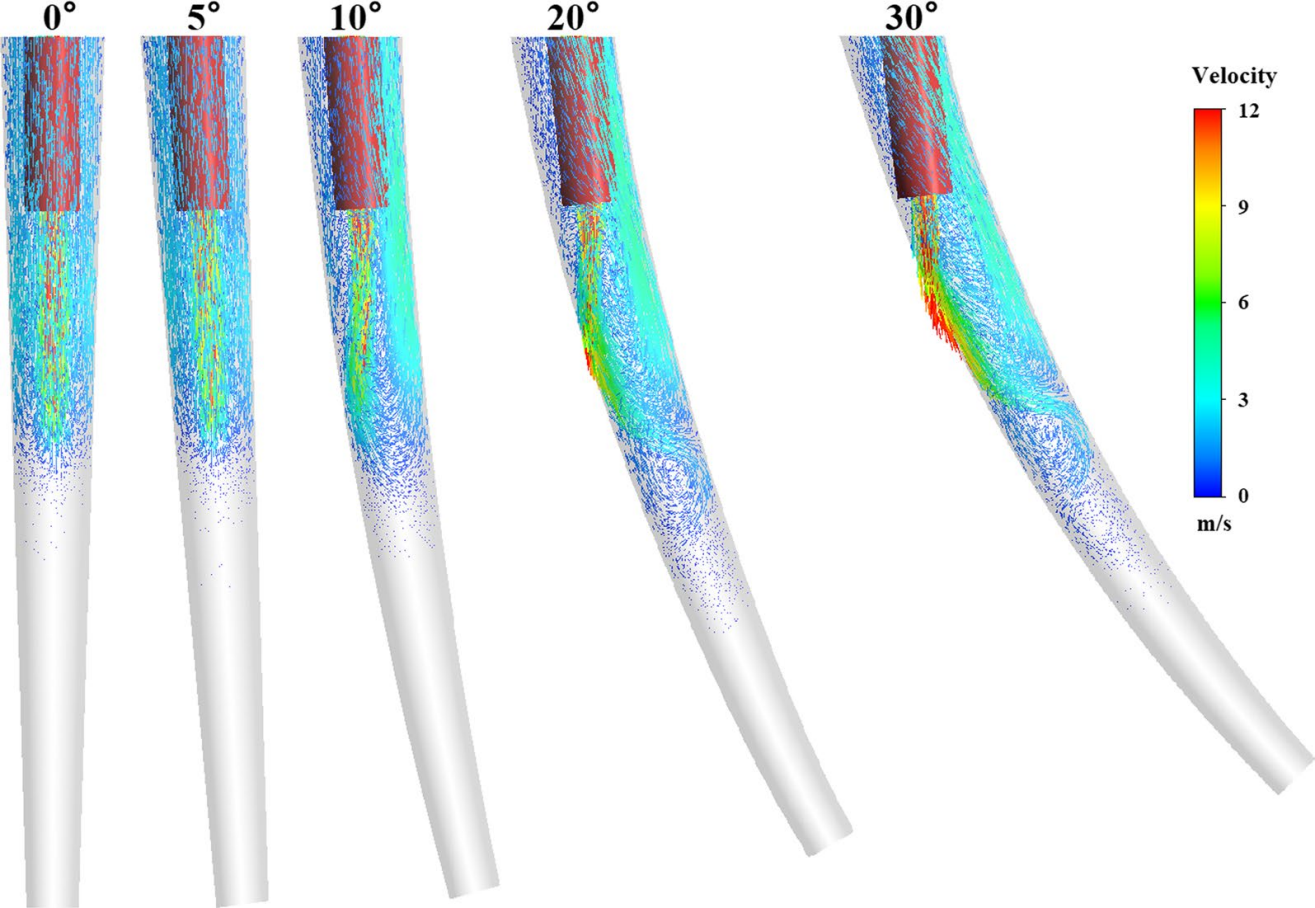
Effect of curvature on the irrigation flow field

The irrigation flow field was modified due to the curving effect of the real root canal. The optimal needle working length of 4.75 mm, which attained maximized clean depth and clean span, was adopted for the differently curving root canal model. Figure 5 shows the flow field structures when we adopted different curvatures of the root canal to explore the effect of the curvature. The apparent difference in the configuration of the apical flow field near the needle tip was realized between different curvatures. The wall significantly modified the stream directions and reorganize the flow field to form different recirculation zones are formed, particularly at the deflection angle > 10°. In one sense, the wall was directed to the flow field. The flow field reaches closer to the tip of the root canal. The range of the apical stagnant zone was suppressed following an increase in the deflection angle of 20° and 30°. Table 2 shows the effect of root canal curvature on irrigation efficiency at the same needle working length. At the deflection angle of 20°, maximum depth of effective velocity was obtained. At the deflection angle of 30°, maximum clean span and clean depth of effective shear stress were obtained.

**Table 2:** Effect of root canal curvature on irrigation efficiency in modified root canals with a 30-G flat needle at 4.75 mm needle working length

Root canal curvature (mm)	Depth of effective velocity^*^ (mm)	Effective shear stress^#^	
		Clean span (mm)	Clean depth (mm)
0°	8.62	2.06	8.37
5°	8.58	1.94	8.35
10°	8.83	2.79	8.40
20°	9.31	3.17	8.77
30°	9.30	3.92	8.86

*Effective velocity: > 0.1 m/s; ^#^effective shear stress: ≥ 100 Pa

**Fig. 6:**
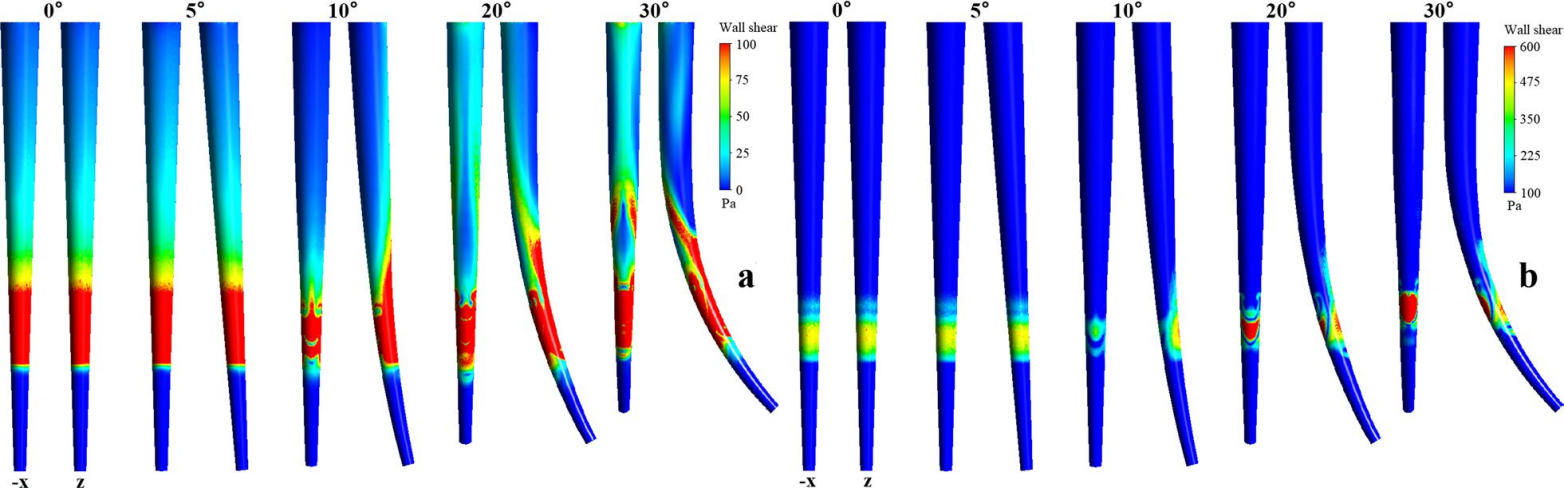
Effect of curvature on stress shear, displayed in the -x and z directions respectively **a** 0–100 Pa; **b** 100–800 Pa

Moreover, the wall shear stress also increased with the curvature owing to the decrease in flushing angle of the irrigation flow against the wall. Figure 6 shows the contour of the shear stress on the wall, the contours of shear stress 0–100 Pa and > 100 Pa were displayed in the -x and z directions. There were noticeable differences in the distribution of the shear stress, including the position and the range between different curvatures. The effective shear stress of 5° was mostly located near the needle tip showing nearly symmetric annular distribution. However, the magnitude of the wall shear stress and the coverage of the effective shear stress was significantly improved following an increase in the curvature or the reflection angle. Additionally, an apparent asymmetric distribution of the effective wall shear stress were found at the reflection angles of 10°, 20°, and 30°, separately. Consequently, Table 2 shows that the effective clean depth and span of the irrigation were improved correspondingly. This implies that curving at the tip of the root canal can help improve the clean efficiency.

Table 3 shows a comparison of irrigation efficiency between real root canal and modified canals at the same needle working length. In real root canal with a curvature of 23.4°, the depth of effective velocity, the clean span, and the clean depth were higher than those of modified root canals with a curvature of 20°or 30°. In the real root canal, the actual human root canal anatomy, including anomalous wall, concave and convex were remained in the inner wall (Fig. 1b). While in the modified canals, the inner wall of root canal was smoothed and modified. The result implies that the minor concave and convex structure improves irrigation efficiency.

**Table 3:** Comparison of irrigation efficiency between real curved canal and modified curved canal

Curved root canal	Depth of effective velocity^*^ (mm)	Effective shear stress^#^	
		Clean span (mm)	Clean depth (mm)
Real 23.4°	9.50	4.96	9.04
Modified 20°	9.31	3.17	8.77
Modified 30°	9.30	3.92	8.86

Real 23.4°: Real canal with a curvature of 23.4°; Modified 20°: Modified canal with a curvature of 20°; Modified 30°: Modified canal with a curvature of 30°

*Effective velocity: > 0.1 m/s

^#^Effective shear stress: ≥ 100 Pa

## Discussion

The application of CFD provides new insights and methods for researchers to explore endodontic treatment. Current CFD researches are mainly focused on the simplified artificial root canal, which is a straight conical pipe with a specific taper. Except for some clinical experiments and in vitro studies [22, 28], no study has investigated the real human root canals to establish whether the shape and the surface of the root canal have a significant impact on the irrigation flow and the clean efficiency. The bending of the real root canal majorly differentiates it from the simplified straight conical root canal model. The needle working length and the structural change of the flow field that stems from the bending curvature can significantly influence the final clean efficiency of the root canal irrigation. The micro-CT image technology allows for the recording of the shapes and the structures of the root canal in a series of cross-sectional layers. Subsequently, an accurate real root canal model can be reconstructed via the reverse reconstruction technology.

The present study explored the effect of the needle working length, the bending curvature and the anomalous walls on the irrigation flow field in a real root canal using the low cost and the widely used clinical flat needle [29]. We analyzed the influence on irrigation based on the obtained flow field. The results demonstrated that the irrigation flow field in the real root canal differs from that in the simplified root canal model [6, 8].

Moreover, the needle working length can effectively influence the irrigation efficiency and cleanability. Under the interaction of root canal curving effect, the different needle working length significantly modify the configuration of the irrigation flow field. Such a phenomenon is different from that in the simplified straight conical canal model. Previous studies revealed that the change of needle working length in the straight conical canal could not necessarily improve the irrigation fluid replacement and transportation at the apical root canal [9]. The velocity vector and streamline of the irrigation flow in the real root canal show, the interaction between the curving wall and the irrigation flow changes with the needle working length. As such, so that the structure of the flow field is modified. The direct flushing against the wall and the recirculation zone influences shear stress on the wall and the effective velocity for replacement. Meanwhile, the clean depth is modified. Herein, the results of the real root canal showed that the maximum clean depth of the effective displacement velocity, the maximum clean depth of the effective shear stress on the wall, and the largest span of the effective shear stress on the wall correspond to different inserted depth, separately. Generally, irrigation with a needle working length of 5 mm or 5.25 mm allows for the large depth of the effective displacement velocity and effective shear stress with low apical pressure. The needle at these positions could optimize the replacement and transportability of the irrigant. Regardless of the needle working length, the cleaning and replacement capabilities could be maintained within a 1 mm range from the needle tip. Therefore, an appropriate needle working length is crucial in the optimization of irrigation efficiency and cleanability.

To be the real root canal, the bending curvature can also directly affect either the cleanability on the wall or the replacement efficiency. Such bending effect is attributed to the modification of the flow field, for example, the average velocity in the cross-section and the wall shear stress. Previous studies on the irrigation of bending root canals mainly focused on clinical and in vitro experiments. Therefore, data on the intrinsic clean mechanism of the irrigation flow is lacking [22, 23]. In this paper, the effect of the curvature on the flushing jet inside the root canal is explicitly displayed. The basic configuration of the flow field is modified when different bending curvatures are adopted. Furthermore, the symmetrical distribution of both the wall shear stress and the flow structure in straight pipe changes to the asymmetrical distribution in bending root canals. A larger bending curvature implies greater modification. Meanwhile, as the root canal is curving, the direct flushing against the wall directly improves the wall shear stress and the cleanability. In the case of the 0° and 5° curvature, no flushing flow against the wall can be observed. The reflection flow back from the wall is somehow weak because of the little flushing angle in the case of the 10° curvature. Notably, an evident recirculation zone can help improve the replacement. However, as the curvature is improved to 20° and 30°, the flow reflected from the wall is strong and produces a multi-recirculation zone with intense turbulence. Such modification of the flow can inevitably improve the mixing, replacement of the local fluid and the wall shear stress. We concluded that the curving at the tip of root canal has no negative effect on the clean efficiency. Also, the severe curvature, or large reflection angle, for example, 20° and 30° to some extent, can improve the irrigation efficiency. This is vital in the efficiency and the range of the replacement of the residual fluid and the clean ability on the wall. Additionally, at the same needle working length, the coverage and the intensity of the wall shear stress at the apical root canal are significantly improved with the curvature. Thus, curving at the apical third of the root canals can improve the irrigation efficiency and clean ability, it not only improves the removal area and efficiency for the smear layer but also better eliminates resistant biofilms.

The elimination of smear layer and debris is the primary goal of endodontic treatment in infected canals [30]. Mechanical shaping and chemical irrigation is the main way of endodontic cleaning. However, mechanical preparation in curved canals may lead to procedure errors such as ledge, zip, perforation. What is more, canals with curvatures located at the apical orifice has been shown higher risks of cyclic fatigue of rotary files [31, 32], which may lead to instrument separation, result in unfavorable prognosis. In this research, it showed that the combination with canal curvature and needle working length can improve irrigation efficiency by increasing irrigation area and shear stress. Thus, in curved canals, it becomes even more important for effective intracanal irrigation. Irrigation serves several purposes: lubricate canals, dissolve the pulp remnants, wash out debris created by instrumentation, eliminate the endodontic pathogens, and clean the dentin surface covered by smear layer. In the apical third of unprepared or not-shaped canals, of which irrigant can not flood the physical apical foramen area with a 30-G flat needle, it is suggested to shake the irrigant inside the canal with 2–3-mm amplified movement using a K-file to reduce the vapor lock effect.

In conventional root canal treatment, the procedure of endodontic cleaning and shaping is a loss of hard tissue, leading to the weakening of the treated tooth [33, 34]. Less invasive endodontic strategies are suggested to be used in endodontic treatment [35]. Irrigation is a feasible way to remove debris while preserve dentin. In this research, a root canal of taper 0.04, apical size #15 was selected. As far as the authors knows, this is the first canal model with minimally invasive prepared canal model, which is quite common in the early stage of root canal treatment. The 30-G flat needle, is widely used because of its cheapness. This research described the movement of irrigant particles in the unshaped canals, which helps us to find a way to retain hard tissue while removing off the debris.

Due to the technical limitations, we have not been able to change the root canal curvature while retaining the minor concave and convex structure on the canal wall. Therefore, when exploring the effect of root curving, it was a modified canal model with smoothing and optimizing inner wall although other parameters remained the same. Interestingly, it was found that with the same needle working depth, in a real canal with a curvature of 23.4°, the irrigation efficiency was reinforced compared with a modified canal with a curvature of 20°or 30°. It implies that the inter-canal rough surface may play a greater role than root curvature in increasing irrigation efficiency. To investigate the principle of this phenomenon, it maybe that lots of vortexes are generated when the micro-stream strikes concave and convex, when all the vortexes collide together, bigger streams were produced. In this study, it was just a physical process. While in clinical practice, sodium hypochlorite produces bubbles when resolving organic matter, the effect of unsteady current on shear stress will be strengthened again with the bubbles. In the future research, a combination of CFD simulation and verification in real teeth was expected to explore the track of irrigant particles, through this way, a more convincing constructive suggestions may be obtained for clinical endodontic irrigation.

## Conclusions

In this work, through micro-CT imaging, a model of a real root canal with taper 0.4, size 15# was constructed. This was followed by a CFD analysis of irrigation of the real root canal. The effect of the needle working length and the bending curvature of the real root canal on the irrigation efficiency and cleanability are then analyzed based on: (i) the effective transport velocity in the cross sections for the replacement of the residual irrigation fluid; (ii) the effective wall shear stress for removing the smear layer on the wall. The following conclusions can be drawn:

1. Due to the coordination of the needle working length and the curving effect of the real root canal, the needle working length can affect the range of stagnant dead water zone, the structure of the flow field near the nozzle of the needle, the distribution of the wall shear stress in the real curving root canal. The depth of the effective wall shear stress decreases monotonically with the increasing working length. While the depth of the effective velocity and the span of the effective wall shear stress changes non-monotonically with the needle working length.
2. The bending curvature of the root canal can modify the structure of the irrigation flow field. Curving at the tip of the root canals can improve irrigation efficiency and cleanability.
3. The anomalous root canal wall enhances endodontic irrigation efficiency.

## Data Availability

The datasets generated during and analyzed during the current study are not publicly available due to their containing information that could compromise the privacy of research participant, but are available from the corresponding author on reasonable request.

## Abbreviations

Micro-CT: Micro-Computed Tomography
CFD: Computational fluid dynamics
D_ext_: External Diameter
D_int_: Internal Diameter
L: Length
